# Genetic Diversity of Porcine Circovirus Isolated from Korean Wild Boars

**DOI:** 10.3390/pathogens9060457

**Published:** 2020-06-09

**Authors:** Sok Song, Gyu-Nam Park, SeEun Choe, Ra Mi Cha, Song-Yi Kim, Bang-Hun Hyun, Bong-Kyun Park, Dong-Jun An

**Affiliations:** 1 Virus Disease Division, Animal and Plant Quarantine Agency, Gimchen, Gyeongbuk-do 39660, Korea; ssoboro9@gmail.com (S.S.); changep0418@gmail.com (G.-N.P.); ivvi59@korea.kr (S.C.); rami.cha01@korea.kr (R.M.C.); topiary2102@daum.net (S.-Y.K.); hyunbh@korea.kr (B.-H.H.); parkx026@korea.kr (B.-K.P.); 2 College of Veterinary Medicine, Seoul University, Gwanak-ro, Gwanak-gu, Seoul 08826, Korea

**Keywords:** PCV2, wild boar, recombination, tMRCA, genotype shift

## Abstract

In Korea, three genotypes of porcine circovirus type 2 (PCV2a, PCV2b, and PCV2d) have been identified on domestic pig farms, while two genotypes (PCV2a and PCV2b) have been identified in wild boar populations. Here, we investigated genotype diversity and genotypic shift in 91 PCV2 isolates from 1340 wild boars captured in South Korea between 2013 and 2017. Phylogenetic analyses based on the complete ORF2 showed that the 91 PCV2 strains were detected as four genotypes by qPCR screening assay: PCV2a (2.2%, 2/91), PCV2b (16.5%, 15/91), PCV2d (80.2%, 73/91), and PCV2h (1.1%, 1/91). Only one intergenotype recombinant event was detected between PCV2 ORF2 in wild boars (PCV2b) and domestic pigs (PCV2a). Amino acid positions 86–89 within ORF2, which distinguishes the different genotypes, were conserved in all PCV2 genotypes isolated from South Korean wild boars, including TNKI in PCV2a/PCV2h, SNPR in PCV2b, and SNPL in PCV2d. The estimated nucleotide substitution rates in the ORF2 region of viruses from South Korean wild boars and domestic pigs were 5.8145 × 10^−4^ and 4.5838 × 10^−4^ substitutions per site per year (s/s/y), respectively. The times to the most recent common ancestor (tMRCA) for South Korean domestic pig PCV2 were 1937 (PCV2a), 1972 (PCV2b), 1999 (PCV2d-1), and 2000 (PCV2d-2). By contrast, the tMRCA for South Korean wild boar PCV2b and PCV2d were 1989 and 2001, respectively. Thus, the PCV2d genotype is prevalent among South Korean wild boars and domestic pigs.

## 1. Introduction

Porcine circoviruses (PCVs), which belong to the genus *circovirus* within the family *Circoviridae*, are small, non-enveloped, single-stranded circular DNA viruses [1,2]. Pathogenic porcine circovirus 2 (PCV2) is the major causative agent of PCV-associated diseases (PCVADs), including postweaning multisystemic wasting syndrome (PMWS), porcine dermatitis and nephropathy syndrome (PDNS), reproductive disorders, and porcine respiratory disease complex, all of which result in significant economic loss to the swine industry worldwide [3,4,5,6]. Contact with infected wild boars is also a causative factor of PCVADs in domestic pigs [7,8]. The genomes of PCVs contain three open reading frames (ORFs). ORF1 (*rep* gene) encodes the replicase proteins involved in virus replication [9], ORF2 (*cap* gene) encodes the capsid protein [10], and ORF3 encodes a protein with potential apoptotic activities [1,2,11,12,13]. Many studies have shown that the ORF2 region of PCV2 is a useful marker for monitoring genetic variation and for phylogenetic studies [2,4,6,14]. Two genotypic shifts in PCV2 occurred during the 20th century—one in 2005 shifted PCV2a with PCV2b, and another in 2008 shifted PCV2b with PCV2d [2,15,16,17]. Phylogenetic analyses of the complete genome and ORF2 region of PCV2 isolates worldwide have shown that PCV2 could be divided into eight distinct genotypes (PCV2a-h) according to a new genotyping method [18]. PCV2d has been shown to have two subgenotypes (2d-1 and 2d-2) [2,19] and the presence of PCV2c has only been reported in Denmark and Brazil [19,20,21], while PCV2e has been reported in China, Thailand, USA, and Mexico [22,23,24,25]; and PCV2f has been reported in China and India; PCV2h has been reported in China and Vietnam; and PCV2g has been reported in China and Ukraine [26,27]. In recent years, phylogenetic analyses based on the complete genome in China has shown diversity in PCV2 strains (PCV2a, PCV2b, PCV2d, PCV2e, and PCV2h) [28]. The genotypic shift from PCV2b to PCV2d also occurred in South Korean domestic pigs [29]; however, the PCV2d genotype was not detected among South Korean wild boars from 2010 to 2012 [30]. Seroprevalence studies and genetic characterization of PCV2 in wild boars have been reported in several countries (South Korea, Hungary, Germany, Spain, Uruguay, Romania, and Brazil) [30,31,32,33,34,35,36,37,38]. The genotypic evolution of PCV2 in free-living wild boars and domestic pigs [38] and the inter- and intragenotype recombination in wild boars have also reported [36] 

The aim of this study was to examine the prevalence and genotypic characteristics of PCV2 isolated from wild boars captured in South Korea between 2013 and 2017.

## 2. Results 

### 2.1. PCV2-Positive Rate and Genotypes Detected in South Korean Wild Boars and Domestic Pigs 

**Figure 1:**
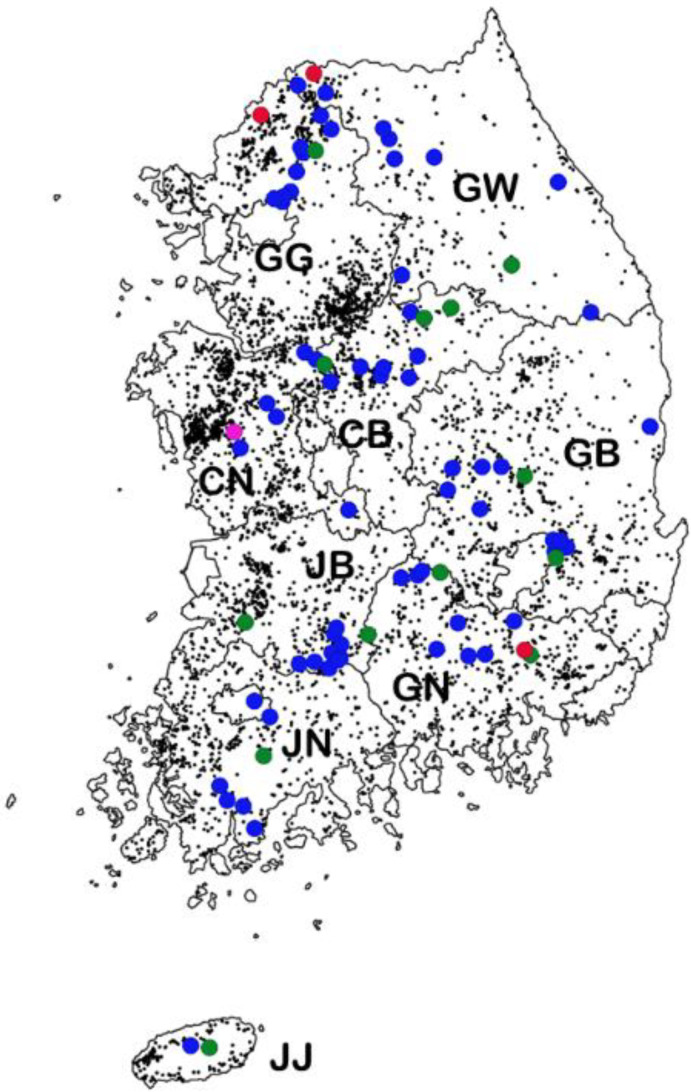
Map showing regions in which wild boars with PCV2 were captured from 2013 to 2017. The locations of pig farms (dark dots) and isolated PCV2a (red dots), PCV2b (green dots), PCV2d (blue dots), and PCV2h (pink dot) strains. GW: Gangwon; GG: Gyeonggi; GN: Gyeongnam; GB, Gyeongbuk; JN: Jennam; JB: Jenbuk; CN: Chungnam; CB: Chungbuk; JJ: Jeju.

**Figure 2:**
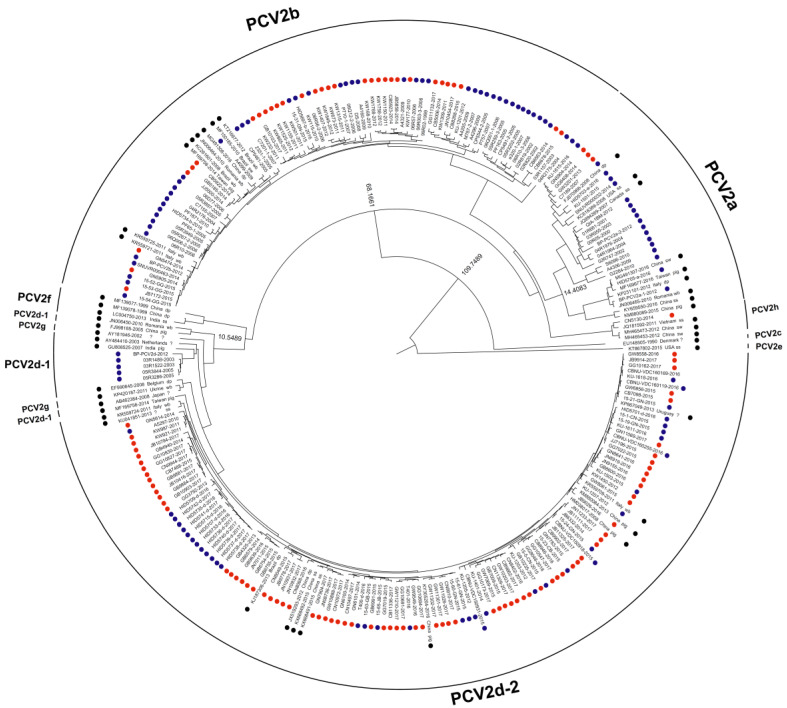
Maximum clade credibility (MCC) trees based on PCV2 ORF2. MCC tree based on 264 PCV sequences (including 91 PCV2 sequences from South Korean wild boars) obtained from the NCBI Genbank database. Each dataset was simulated using the following options: generation = 100,000,000; 10% burn-in; and ESSs > 200. PCV2 strains detected from South Korean wild boars and South Korean domestic pigs are marked with red and blue dots, respectively. PCV2 strains detected from other countries are marked with black dots. Node labels indicate branch times.

**Figure 3:**
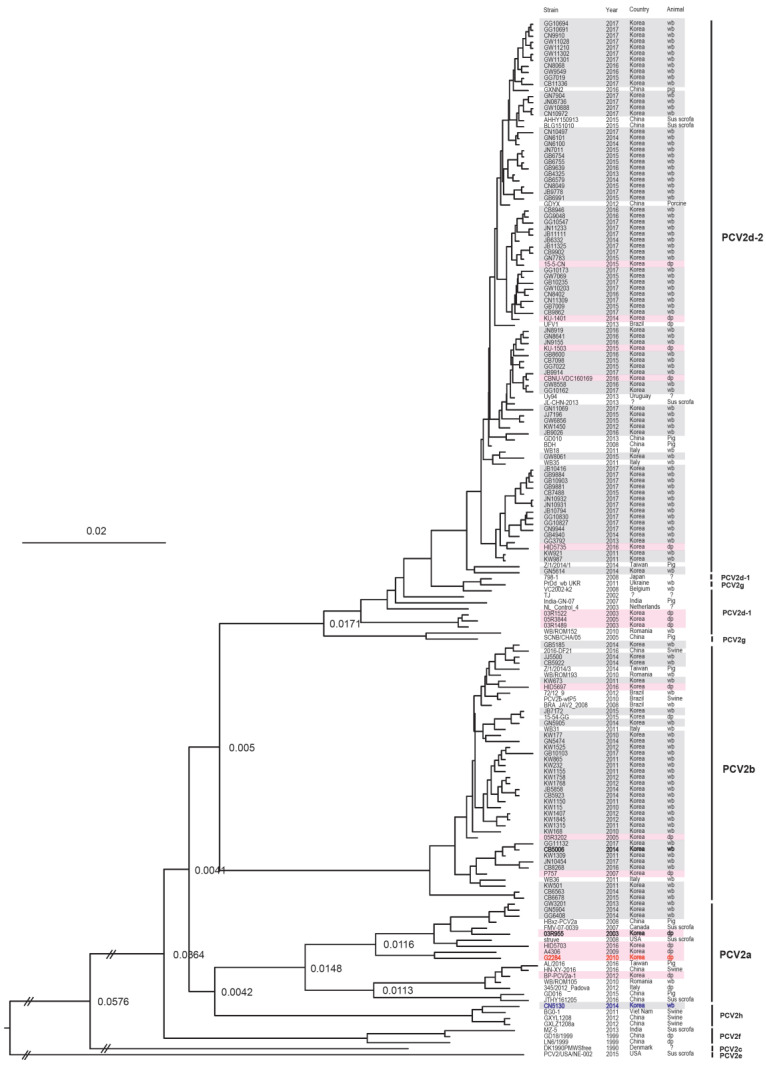
MCC tree based on PCV2 ORF2 sequences from South Korean wild boars. The 110 complete ORF2 gene sequences were obtained 19 South Korean wild boars (2010–2012) from the NCBI GenBank database and 91 South Korean wild boar PCV2 (2013–2017) in this study. The MCC tree was constructed using Tree Annotator v.1.10 in the BEAST software package (University of Auckland, New Zealand), with a 10% burn-in. CN5130 strain (blue color) was contained in PCV2h group with BGO-1, GXYL1208, and GXLZ1208a strains. The recombination of G2284 strain (red color) was found between the major parent (03R955 strain) and the minor parent (CB5006) strain (black color) by the RDP 4 program. PCV2 strains detected from South Korean wild boars and South Korean domestic pigs are marked with gray and pink background colors, respectively. Node labels indicate branch times.

The quantitative real-time PCR (qPCR) revealed that 6.8% (*n* = 91) of 1340 wild boars captured in nine geographical areas between 2013 and 2017 were PCV2-positive. The yearly PCV2-positive rates were 2.3% (3/152) in 2013, 5.7% (18/318) in 2014, 5.6% (17/303) in 2015, 8.2% (13/158) in 2016, and 9.8% (40/409) in 2017 (Table 1). The complete ORF2 nucleotide sequences of the 91 PCV2 isolates from South Korean wild boars captured in this study belonged to four genotypes: 2.2% (2/91) were PCV2a, 16.5% (15/91) were PCV2b, 80.2% (73/91) were PCV2d, and 1.1% (1/91) were PCV2h (Table 1 and Figure 1, Figure 2 and Figure 3). 

The PCV2-positive rates according to geographical region over the 5 year period (2013–2017) were as follows: 7.0% (12/172) in Gangwon (GW); 7.7% (13/169) in Gyeonggi (GG); 5.9% (12/203) in Gyeongnam (GN); 5.6% (13/234) in Gyeongbuk (GB); 6.2% (8/129) in Jennam (JN); 12.5% (11/88) in Jenbuk (JB); 5.6% (10/179) in Chungnam (CN); 6.3% (10/158) in Chungbuk (CB); and 25% (2/8) in Jeju (JJ) (Figure 1). 

### 2.2. Rates of Nucleotide Substitution per Site per Year and the Time to the Most Recent Common Ancestor

We used a Bayesian MCMC approach to estimate the rate of nucleotide substitution per site per year (s/s/y) and the time to the most recent common ancestor (tMRCA) for 264 PCV2 strains. Of the 264 PCV2 strains examined, 91 were isolated from South Korean wild boars in this study, 19 from South Korean wild boars captured between 2010 and 2012, and 109 from South Korean domestic pigs (1999–2017; data for 2010–2012 (wild boars) and 1999–2017 (domestic pigs) were obtained from GenBank). The estimated nucleotide substitution rates in the ORF2 region of PCV2 from South Korean wild boars and domestic pigs were 5.8145 × 10^−4^ (s/s/y) and 4.5838 × 10^−4^ (s/s/y), respectively (Table 2). The tMRCA values for South Korean domestic pig PCV2a, PCV2b, PCV2d-1, and PCV2d-2 were 1937 (95% highest posterior density (HPD) lower–upper: 1916–1956), 1972 (95% HPD: 1957–1987), 1999 (95% HPD: 1994–2003), and 2000 (95% HPD: 1993–2005), respectively (Table 2 and Figure 3). A phylogenetic tree revealed that PCV2 from South Korean domestic pigs clustered into three genotypes: PCV2a, PCV2b, and PCV2d (Figure 2 and Figure 3). The tMRCA for South Korean wild boar PCV2b and PCV2d was 1989 (95% HPD lower–upper: 1983–1996) and 2001 (95% HPD: 1994–2007), respectively (Table 2 and Figure 3).

### 2.3. Bayesian Skyline Plot Analysis

**Figure 4:**
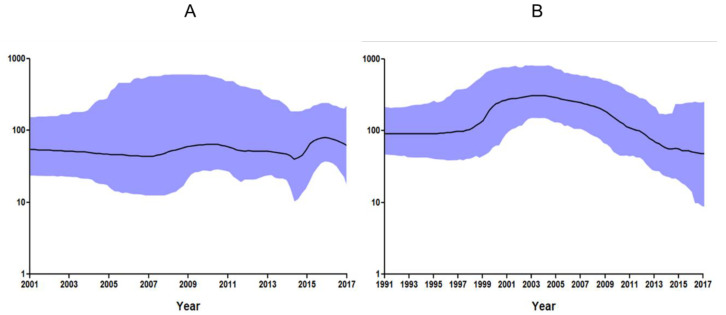
Bayesian skyline dots for the complete PCV2 ORF2 gene. The x-axis shows the years before 2017, while the y-axis represents the effective size of the viral population in South Korean wild boars (**A**) and domestic pigs (**B**). The black line represents the median estimate of the effective number of infections over time, while the blue shading indicates the 95% highest posterior density (upper and lower boundaries).

The phylodynamics of PCV2 isolated from South Korean wild boars and domestic pigs were estimated using a Bayesian skyline plot (BSP) based on the ORF2 (capsid protein) gene. The effective population size (median) of South Korean wild boar PCV2 strains was stable (between 40.1 and 70.3) from 2001 to 2017, although it increased a little between 2015 and 2016 (Figure 4A). The effective population size (median) of PCV2 in South Korean domestic pigs was very high (between 201.7 and 305.4) between 1999 and 2008, but fell after 2009; the median effective population size in 2017 was 49.5 (Figure 4B).

### 2.4. Amino Acid Sequence Analysis of the PCV2 ORF2 Protein from South Korean Wild Boars and Domestic Pigs

The nucleotide and amino acid sequences of PCV2 ORF2 strains aligned using the CLUSTAL X alignment program [39]. The nucleotide (nt) and amino acid (aa) sequence identity between each genotype isolated from South Korean wild boars was 89.6–99.7% and 89.4–100%, respectively, whereas that between strains from South Korean domestic pigs was 88.8–99.4% and 86.8–99.6%, respectively. A commercial PCV2 vaccine based on the PCV2a strain was manufactured. Among PCV2 strains isolated from South Korean wild boars, the amino acid sequences at position 86–89, which is thought to distinguish between PCV2a and PCV2b, were 86-TNKI-89 (n = 3) for PCV2a and PCV2h; 86-SNPR-89 (n = 31) for PCV2b and 86-SNPL-89 (n = 76) for PCV2d (Table 3). AA residues important for antibody recognition (residues 173–175 and 179) were conserved (173-YFQ-175 and 179-K) in all genotypes isolated from South Korean wild boars and domestic pigs (Table 3). AA positions 190–191, 206, and 210, which are important for PCV2 replication in vitro, were conserved only in PCV2d (190-TG-191, 206-I, and 210-D) isolated from South Korean wild boars; other genotypes showed sequence variations in both South Korean wild boars and domestic pigs (Table 3).

### 2.5. Recombination Analysis Using Recombination Detection Program

One intergenotype recombinant was detected in ORF2 gene between the PCV2a of domestic pigs and PCV2b of wild boars. G2284 strain detected from South Korean domestic pigs in 2010 was selected as recombinant and the breakpoint of recombination was determined by MaxiChi, Chimaera, SIScan, and 3Seq methods (*p* < 0.01). The beginning and ending breakpoints probability of recombination were determined for positions 4 and 339 in alignment. The major parent was the 03R955 strain detected from South Korean domestic pigs in 2003 and the minor parent was the CB5006 strain detected from South Korean wild boars in Chungbuk (CB) region in 2014. No intragenotype recombinant event was detected in wild boars and in domestic pigs.

## 3. Discussion

Previous studies suggest that PCV2 circulates at a high rate among wild boar populations in Europe; indeed, it is speculated that these animals might serve as important reservoirs for PCV2 [31,33,34]. In particular, genotype PCV2d-2, which infects both domestic pigs and wild boars, was reported in Italy [35]. PCV2a and PCV2b genotypes were circulating within wild boars in Romania [40] and PCV2b was also detected in wild boars in Uruguay [38]. Our results showed that PCV2d has been detected in wild boars in mountains nationwide in South Korea; since 2015, the main genotypes of PCV2 that have been detected are PCV2d and PCV2b (minor levels), but no PCV2a has been detected. PCV2a, PCV2b, and PCV2d were first isolated from South Korean wild boars in 2013, 2010, and 2011, respectively. Interestingly, the CN5130 strain detected in Korean wild boars in 2014 was contained within the PCV2h group in Vietnam (BGO-1 strain) and China (GXYL1208, and GXLZ1208a strains).

In South Korean wild boars, the tMRCA for PCV2b was 1989 and that of PCV2d was 2001. In PCV2 strains from domestic pigs and wild boars in Sardinia, Italy, the tMRCA values for PCV2b, PCV2d-2, and PCV2d were 2005 (2001–2010), 2009 (2006–2012), and 2011 (2011–2012), respectively [35]. The tMRCA values for Taiwanese PCV2a, PCV2b, and PCV2d-2 were also dated to 1970, 1992, and 2004, respectively [41]. In another large-scale epidemiological study, the tMRCA values for PCV2a, PCV2b, and PCV2d were from 1964, 1973, and 1958, respectively [14].

In South Korean wild boar, the tMRCA values were 1989 (PCV2b) and 2001 (PCV2d), whereas in domestic pigs these were 1972 (PCV2b), 1999 (PCV2d-1), and 2000 (PCV2d-2). This may be because the rate of genetic evolution in wild boars (5.8145 × 10^−4^ s/s/y) is slower than that in domestic pigs (4.5838 × 10^−4^ s/s/y). Another reason may be that genetic recombination in wild boars is lower than that in domestic pigs due to the reduced population density experienced by the former. The substitution rates of PCV2 in South Korean wild boars (5.8145 × 10^−4^ s/s/y) and domestic pigs (4.5838 × 10^−4^ s/s/y) reported herein are slightly lower than those reported in previous studies (2.93 × 10^−3^ s/s/y and 1.44 × 10^−3^ s/s/y, respectively) [2,42], but are similar (8.467 × 10^−4^ s/s/y) to that reported for Taiwanese PCV2 [41].

A BSP showed that the effective population size (median) of PCV2 in South Korean wild boars was stable (between 40.1 and 70.3) from 2001 to 2017. Over the past 10 years, the number of wild boars in South Korea has been estimated to be about 350,000 (250,000–450,000); the lack of rapid population increase suggests that the PCV2 levels have remained constant. However, the increase in the effective population size (201.7–305.4) of South Korean domestic pigs from 1999 to 2008 led to sharp increases in the incidence of PMWS and PCVAD [29].

ORF2-based intra- and intergenotype recombination in wild boar populations and the possible recombination between PCV2 strains of wild boars and domestic pigs were detected 5 cases [33]. In Uruguay, PCV2 recombinant strain circulation revealed a predominance, suggesting that PCV2 recombination can lead to the emergence of strains able to complete and potentially displace parental ones [34]. The different patterns of natural intergenotype recombination found between PCV2 parental strains (PCV2a and PCV2b) in China [13,43,44], USA [45], and South Korea [46]. We first reported the recombination of intergenotypes (PCV2a and PCV2b) between wild boars and domestic pigs in South Korea. However, there seems to be a low probability of current intragenotype and intergenotype recombination within wild boars.

AA residues 86–89 within the ORF2 region of PCV2 are thought to distinguish between PCV2a and PCV2b [47]. In South Korean wild boars, aa positions 86–89 were conserved for TNKI (PCV2a and PCV2h), SNPR (PCV2b), and SNPL (PCV2d). However, this does not hold true in domestic pigs. This may be because the PCV2 capsid protein has been subjected to different selective pressures that act on the different genotypes [14]. Residues 190, 191, 206, and 210 within the capsid protein are crucial for PCV2 replication in vitro [47]. These residues in South Korean PCV2a and PCV2h wild boars were SRID (*n* = 3), while those in PCV2d were TGID (*n* = 75) and SAID (*n* = 1). The residues in most PCV2b isolates from South Korean wild boars were AGIE (*n* = 24); however, changes in residues of PCV2b were noted in seven strains (TGIE (*n* = 5) and AGKE (*n* = 2)), which may have an effect on replication. Residues 173–175 and 179 are crucial for antibody recognition [48]; we found that the aa values in these positions in PCV2a, PCV2b, and PCV2d were conserved (YFQ and K) in South Korean wild boars and domestic pigs. This is consistent with the finding that aa 169–180 of the capsid protein are highly conserved among all PCV2 genotypes [49].

In conclusion, PCV2 strains isolated from South Korean wild boars have four genotypes; of these, PCV2d strains appeared mainly to be from 2015. Each genotype (PCV2a, PCV2b, and PCV2d) in South Korean wild boars seems to have evolved in a stable and independent manner, whereas some strains might show unusual changes, such as PCV2h. The data suggest that the PCV2d genotype will continue to circulate in wild boars.

## 4. Materials and Methods

### 4.1. Sample Collection and qPCR

A total of 1340 whole blood samples were collected from captive wild boars in 9 provinces of South Korea from 2013 to 2017. Total DNA was extracted directly from 250 μL of each blood sample using a DNeasy Blood and Tissue kit (Qiagen Inc., CA, USA) following the manufacturer’s instruction and eluted with 200 μL of elution buffer. For PCV2 detection in extracted viral DNA samples, quantitative real-time PCR (qPCR) was performed on an C1000 thermocycler (BIO-RAD, CA, USA) using the TaqMan-based VDx PCV2 qPCR kit (MEDIAN Diagnostics Inc., South Korea) according to the manufacturer’s instructions. Briefly, amplification was conducted in a 20 μL reaction mixture containing 5 μL extracted DNA and 15 μL TaqMan reaction mixture. The thermal profile for the amplification was 95 for 5 min, followed by 40 cycles of 95 for 10 s and 60 for 40 s. Analysis of each data sample was performed with Bio-Rad CFX Manager software version 3.1 (CA, USA). The cut-off CT value was 35, with values less than 35 considered PCV2 positive. PCV2 positive control DNA was supplied by the manufacturer. The entire ORF2 gene of PCV2 was performed the PCR amplification as describe previously study [50]. All PCR reaction was performed using the AccuPower^®^ ProFi Taq PCR PreMix kit (Bioneer Inc., Daejeon city, South Korea). All purified PCR products were sequenced using the sequencing service of the professional molecular analysis company (CosmoGENTECH Inc., Seoul, South Korea).

### 4.2. Sequence Analysis of the ORF2 Protein

The complete ORF2 nucleotide sequences of 91 PCV2 strains detected from wild boars were submitted to the GenBank under accession numbers MT376295-MT376380 and MT501728-MT501732. The complete ORF2 gene sequences for 173 PCV2 ORF2 (except 91 South Korean wild boar PCV2 in this study) were obtained from the NCBI Genbank database. The 173 PCV2 strains contained 1 PCV2c (EU148505), 1 PCV2e (KT867802), 3 PCV2f (MF139077, MF139078, and LC004750), 2 PCV2g (KP420187 and FJ998185), 3 PCV2h (MH465453, MH465473, and JQ181592), and 20 PCV2 (domestic pigs) from China, Japan, Taiwan, India, Italy, Netherlands, Belgium, Brazil, and Uruguay; 15 PCV2 (wild boars) from China, USA, Canada, Italy, Romania, and Brazil; 109 PCV2 from South Korean domestic pigs (from 1999 to 2017); and 19 PCV2 from South Korean wild boars (from 2010 to 2012). The nucleotide and amino acid sequences of PCV2 ORF2 strains were aligned using the CLUSTAL X alignment program [39].

### 4.3. Phylodynamic and Phylogeographic Analysis

A BEAST input file was generated using BEAUti within the BEAST package v.1.8.1 [51]. The rate of nucleotide substitutions per site per year and the time from the most recent common ancestor (tMRCA) were estimated using a Bayesian MCMC approach. Each dataset was simulated using the following options: generation = 100,000,000; 10% burn-in; and ESSs > 200. The exponential clock and expansion growth population model in the BEAST program was used to obtain the best-fit evolutionary model, while the maximum clade credibility (MCC) tree was visualized using Figtree 1.4 [52].

### 4.4. Recombination Analysis

The recombination between PCV2 from South Korean wild boars and PCV2 from South Korean domestic pigs was analyzed by the recombination detection program (RDP4 version 4.22) [53]. Briefly, this program identifies recombinants from a group of aligned DNA sequences using a number of recombination detection and analysis algorithms. The aligned sequences were evaluated by the RDP, GeneConv, SiScan, MaxChi, BootScan, Chimera, and 3Seq methods in RDP version 4.22, while the highest acceptable result was obtained when the *p*-value was set to 0.01. We have used 264 PCV2 strains for RDP4 analysis. Specifically, we used 91 strains identified from wild boars in this study (2 PCV2a, 15 PCV2b, 73 PCV2d, and 1 PCV2h) and 172 other PCV2 strains obtained from NCBI Genbank database (27 PCV2a, 72 PCV2b, 1 PCV2c, 11 PCV2d-1, 53 PCV2d-2, 1 PCV2e, 3 PCV2f, 2 PCV2g, and 3 PCV2h). Detailed information of all PCV2 strains were indicated in Figure 2.

### 4.5. Ethics Approval

The authors confirm that the work complies with the ethical policies of the journal. The work was approved by the Institutional Animal Care and Use Committee of the Animal and Plant Quarantine Agency (APQA) (Approval Number: 2019-448).

## Figures and Tables

**Table 1:** Genotypic analysis of 219 South Korean porcine circovirus type 2 (PCV2) sequences collected from 1999 to 2017.

Year	South Korean Wild Boars (*n* = 110)				South Korean Domestic Pigs (*n* = 109)			
	PCV2a	PCV2b	PCV2d	PCV2h	PCV2a	PCV2b	PCV2d-1	PCV2d-2
1999	0	0	0	0	0	1	0	0
2000	0	0	0	0	1	0	0	0
2001	0	0	0	0	1	0	0	0
2002	0	0	0	0	1	2	0	0
2003	0	0	0	0	1	1	2	0
2004	0	0	0	0	2	2	0	0
2005	0	0	0	0	0	11	2	0
2006	0	0	0	0	0	9	0	0
2007	0	0	0	0	1	6	0	0
2008	0	0	0	0	0	1	0	0
2009	0	0	0	0	1	5	0	0
2010	0	3	0	0	2	1	0	1
2011	0	8	2	0	0	0	0	0
2012	0	5	1	0	3	2	1	3
2013	1 *	0	2 *	0	0	0	0	0
2014	1 *	9 *	7 *	1 *	1	1	0	1
2015	0	2 *	15 *	0	1	4	0	13
2016	0	1 *	12 *	0	3	2	0	13
2017	0	3 *	37 *	0	0	0	0	7
Total	2	31	76	1	18	48	5	38

* In this study, PCV2 positives were detected in wild boars from 2013 to 2017.

**Table 2:** Substitution rate and tMRCA of different lineages of porcine circovirus (ORF2 gene).

Group	Lineage	Substitution Rate (×10^−4^ Substitutions/Site/Year)	tMRCA ^a^ (95% HPD Lower–Upper)
South Korean wild boars	PCV2	5.8145 (3.3616–8.5714)	
	PCV2a		
	PCV2b		1989 (1983–1996) ^a^
	PCV2d		2001 (1994–2007)
South Korean domestic pigs	PCV2	4.5838 (3.0285–6.1759)	
	PCV2a		1937 (1916–1956)
	PCV2b		1972 (1957–1987)
	PCV2d-1		1999 (1994–2003)
	PCV2d-2		2000 (1993–2005)

^a^ tMRCA: the time to the most recent common ancestor.

**Table 3:** Variable amino acid residues in the putative capsid proteins of 219 South Korean PCV2 strains.

Function	Amino Acid Position	South Korean Wild Boars (*n* = 110)			South Korean Domestic Pigs (*n* = 109)			
		PCV2a (*n* = 2), PCV2h (*n* = 1)	PCV2b (*n* = 31)	PCV2d (*n* = 76)	PCV2a (*n* = 18)	PCV2b (*n* = 48)	PCV2d-1 (*n* = 5)	PCV2d-2 (*n* = 38)
Distinguish between PCV2a & 2b	86–89	TNKI(3)	SNPR(31)	SNPL(76)	TNKI(16) SNPL(1) SNKI(1)	SNPR(48)	SNPL(5)	SNPL(37) SDPL(1)
Important for antibody recongition	173–175, 179	YFQK(3)	YFQK(31)	YFQK(76)	YFQK(18)	YFQK(48)	YFQK(5)	YFQK(38)
Important for PCV2 replication in vitro	190–191, 206, 210	SRID(3)	AGIE(24) TGIE(5) AGKE(2)	TGID(75) SAID(1)	SRKD(10) SRID(7) SKKD(1)	AGIE(43) TGIE(5)	TGID(5)	TGID(29) TGIG(8) TGIE(1)
